# Supplementary material for: The virulence regulator bvgS controls nutrient-induced filamentation in Bordetella avium
Source: J Bacteriol. 2025 Aug 12;207(9):e00030-25. doi: 10.1128/jb.00030-25 (PMC12445083; doi:10.1128/jb.00030-25)
Supplement: Supplemental figures — Fig. S1 to S6. [file jb.00030-25-s0001.pdf]

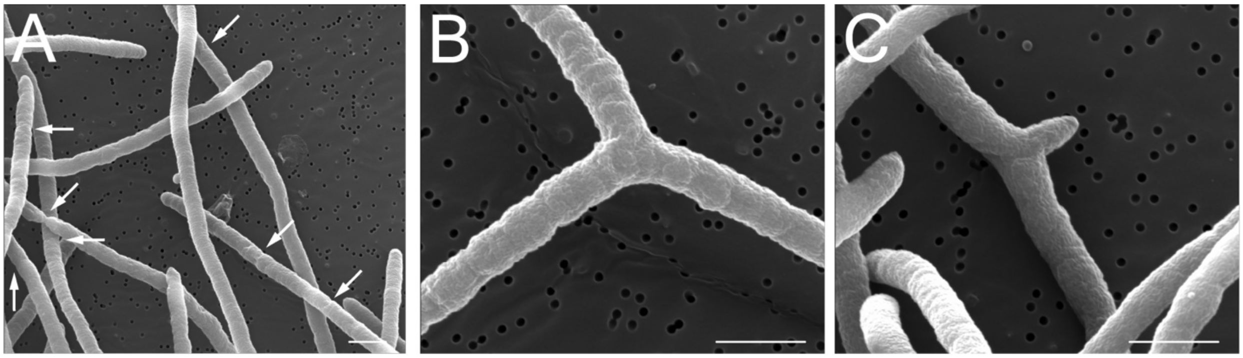

Supplemental Figure 1. A) Filamentous *B. avium* grown in TB showing early signs of septation indicated by white arrows. B-C) Filamentous *B. avium* grown in TB showing branching. Scale bar=1 µm.

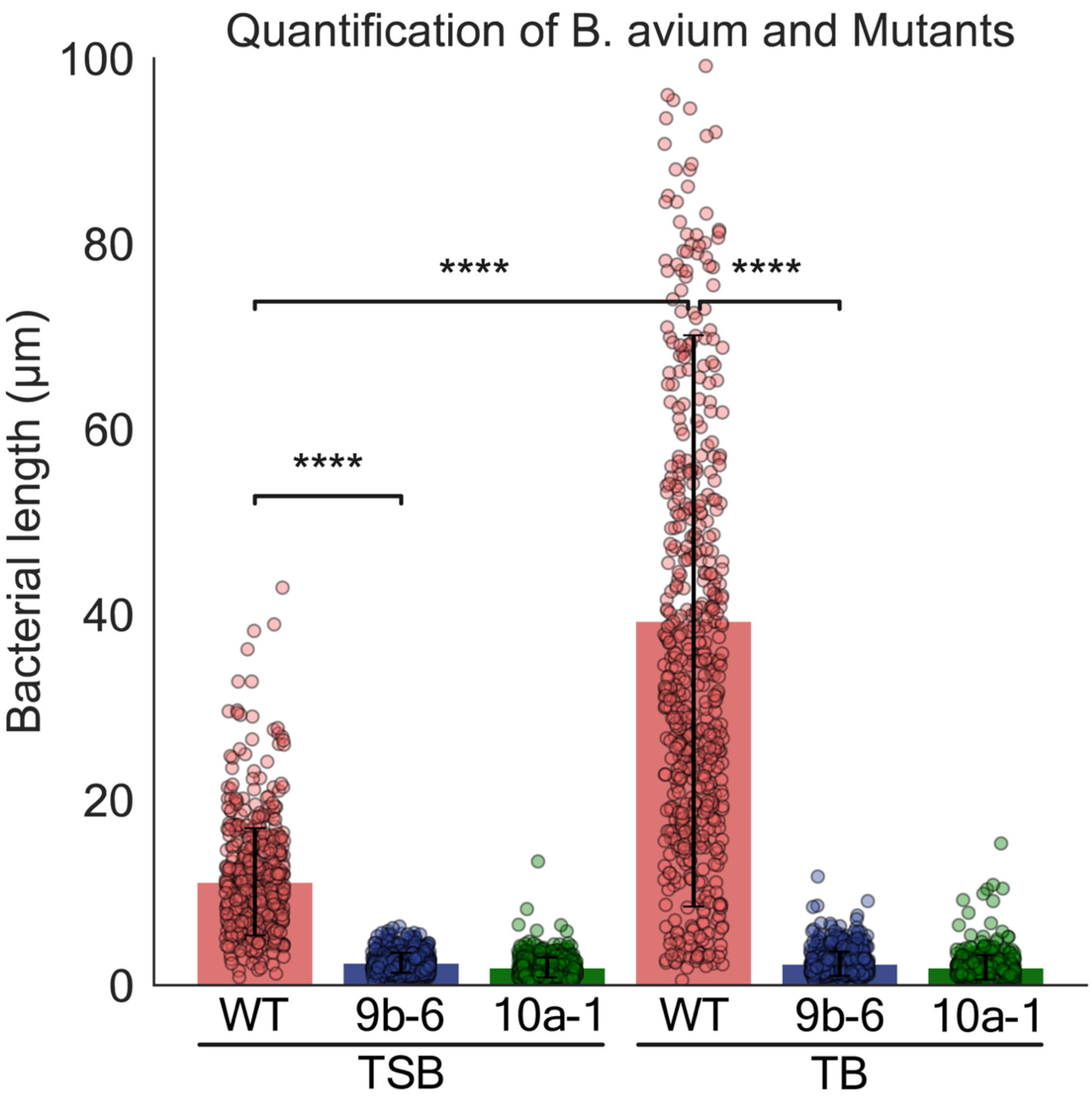

Supplemental Figure 2. Distribution of bacterial cell lengths measured from individual cells of WT *B. avium,* mutant 9b-6, and mutant 10a-1 grown in TSB and TB. Experiments were done in triplicate (N=3), n=200/replicate, p<0.0001 (****), by Mann-Whitney test.

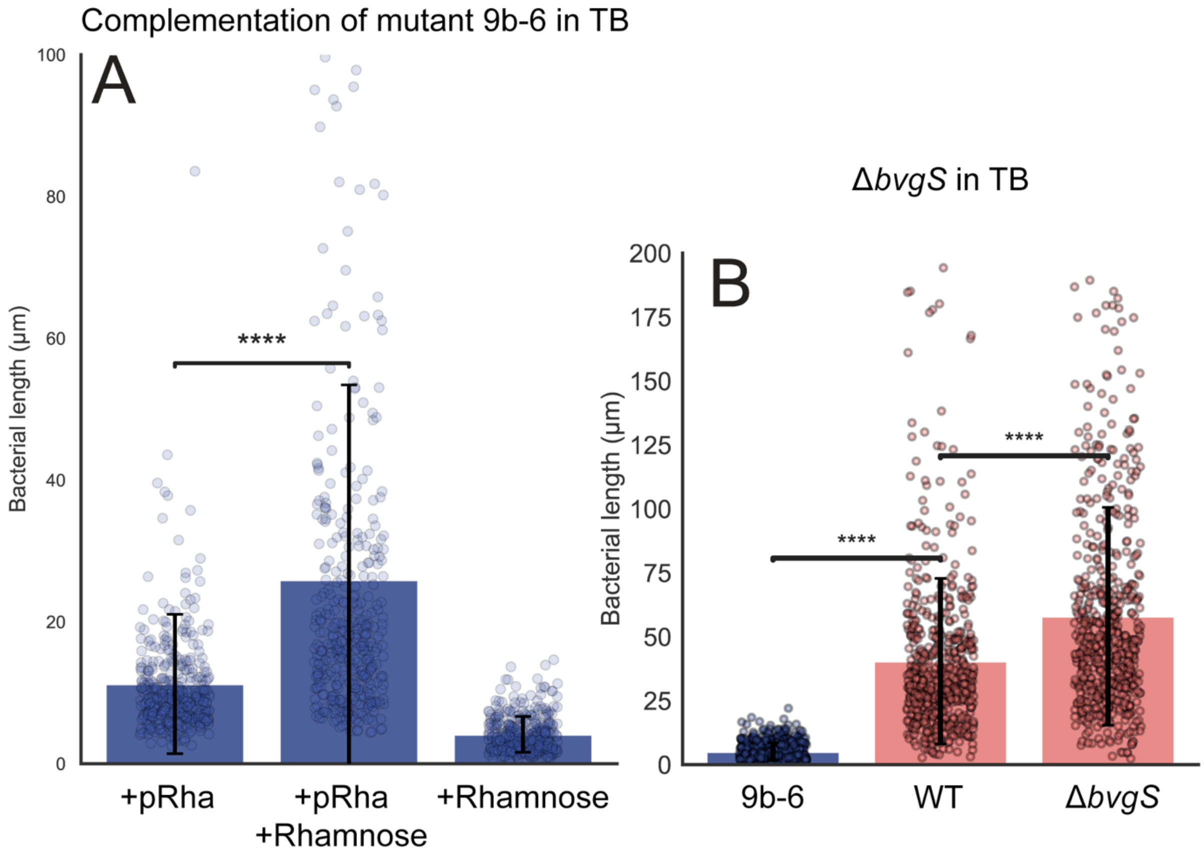

Supplemental Figure 3. A) Distribution of bacterial cell lengths measured from individual cells of non-filamentous mutant 9b-6 complemented with WT *bvgS*, with and without induction of plasmid using rhamnose (200 µg/ml), grown in TB. B) Distribution of bacterial cell lengths measured from individual cells of non-filamentous mutant 9b-6, WT *B. avium*, and Δ*bvgS*, grown in TB. Experiments were done in triplicate (N=3), n=200/replicate, $p<0.0001$ (****), by Mann-Whitney test.

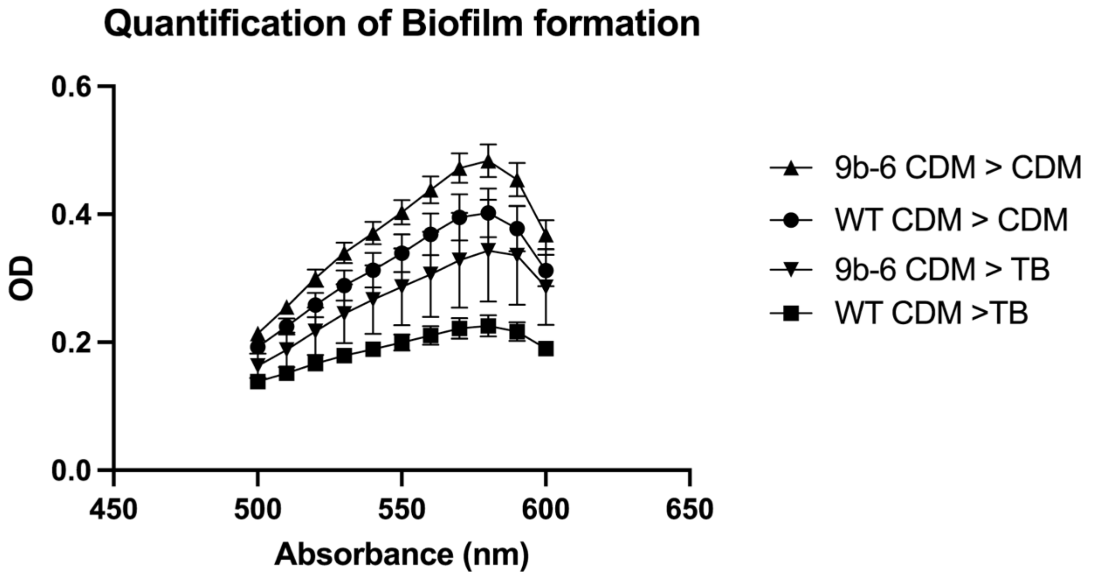

Supplemental Figure 4. Quantification of biofilm formation using crystal violet on non-filamentous mutant 9b-6 and WT *B. avium* grown in CDM and TB. Y-axis shows comperative OD readings, X-axis shows absorbance (nm).

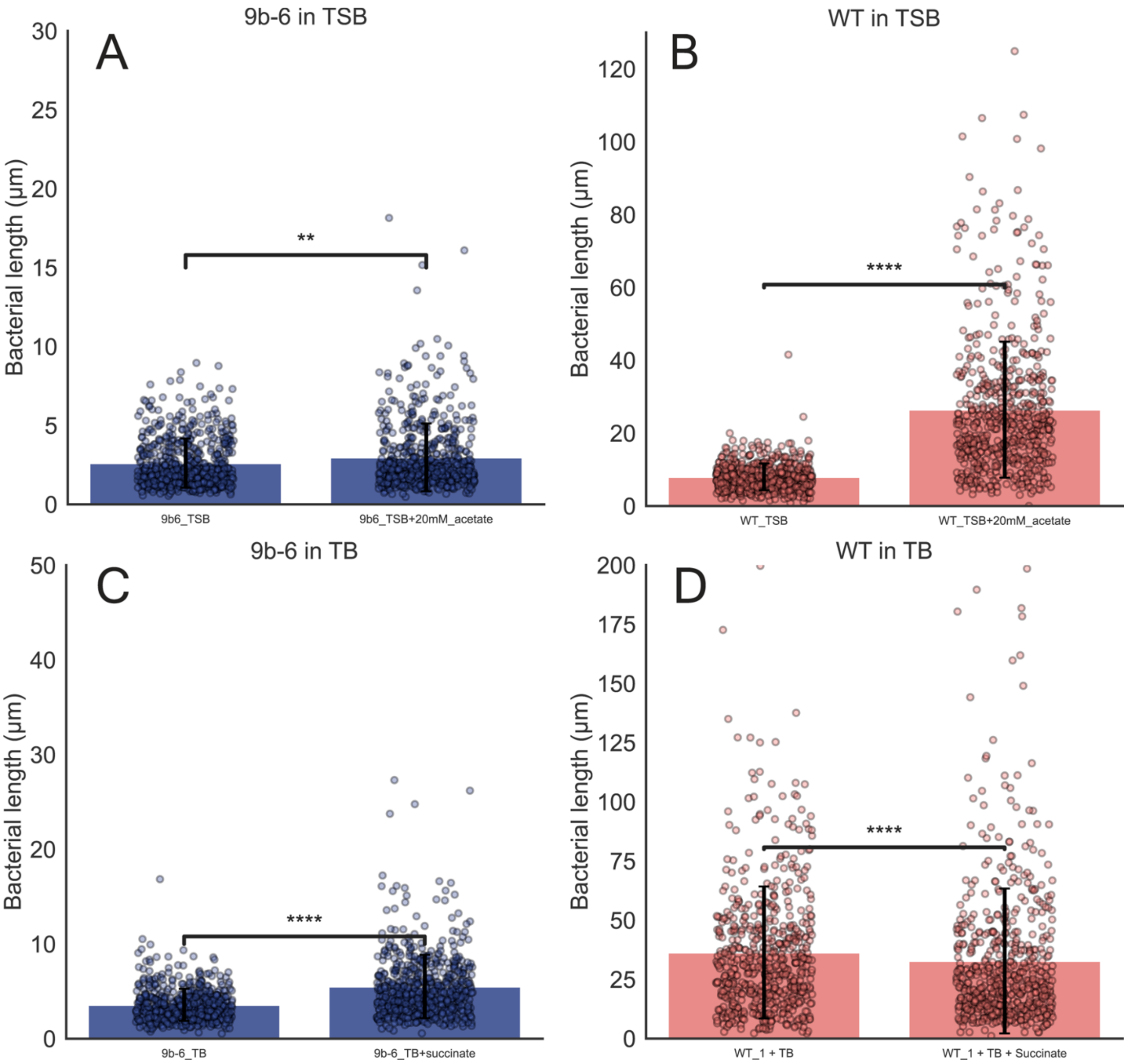

Supplemental Figure 5. A-B) Distribution of bacterial cell lengths measured from individual cells of non-filamentous mutant 9b-6 and WT *B. avium* grown in TSB with and without sodium acetate (20 mM). C-D) Distribution of bacterial cell lengths measured from individual cells of non-filamentous mutant 9b-6 and WT *B. avium* grown in TB with and without succinate (1 mM). Experiments were done in triplicate (N=3), n=200/replicate, $p<0.01$ (**), $p<0.0001$ (****), by Mann-Whitney test.

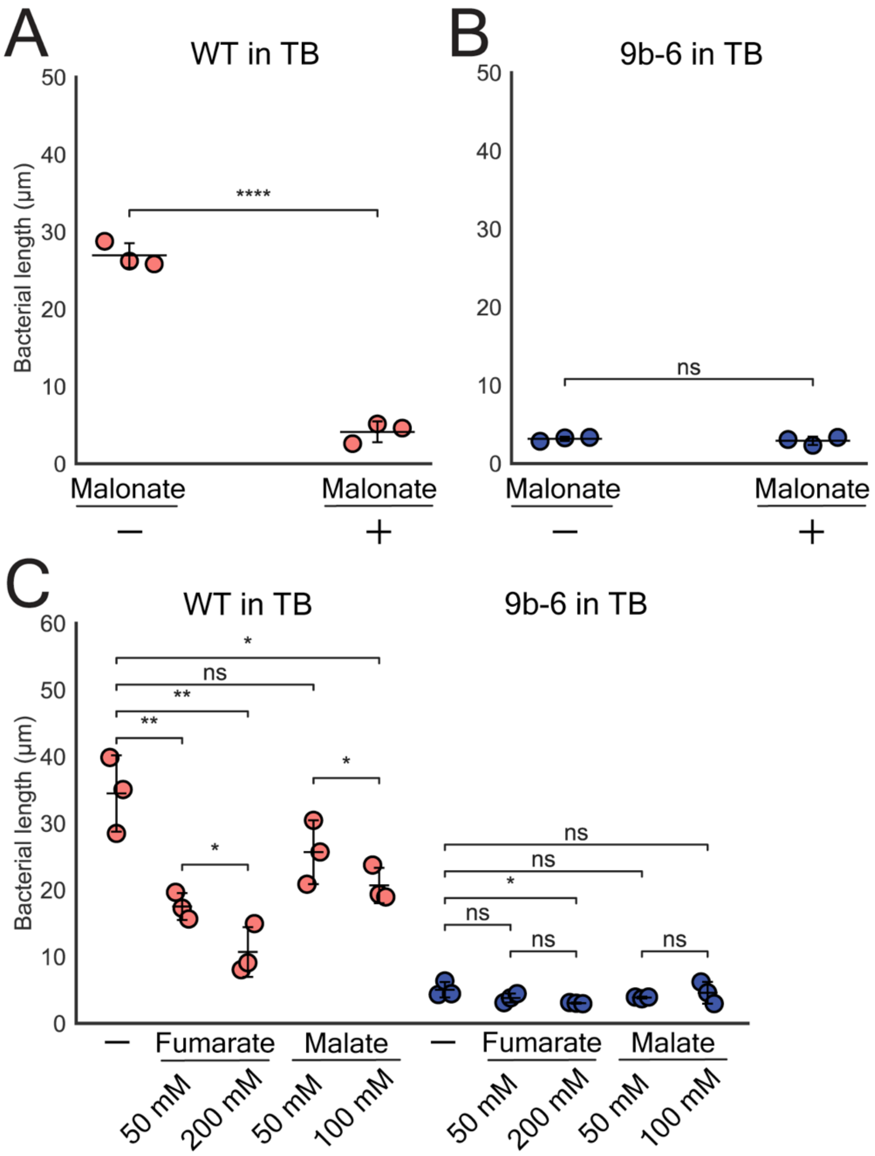

Supplemental Figure 6. A) WT *B. avium* grown in TB with supplemented malonate (80 mM) showing inhibition of filamentation. B) Mutant 9b-6 grown in TB with supplemented malonate (80 mM) showing no change in bacterial length. C) WT *B. avium* in red and mutant 9b-6 in blue, supplemented with fumarate (50, 200 mM) or malate (50, 100 mM). Experiments were done in triplicate (N=3), n=200/replicate, $p<0.05$ (*), $p<0.01$ (**), $p<0.0001$ (****), ns=not significant, by unpaired two-tailed t-test. Error bars represent SD.
